# Molecular docking analysis of amyloid precursor protein with compounds from the Australian cowplant 

**DOI:** 10.6026/97320630016561

**Published:** 2020-07-31

**Authors:** Jayaraman Selvaraj, Hussain Sardar, Veeraraghavan Vishnupriya, Janardhana Papayya Balakrishna, Surapaneni Krishna Mohan, Rajamanickam Pon Nivedha, Periyasamy Vijayalakshmi, Rajagopal Ponnulakshmi

**Affiliations:** 1 Department of Biochemistry, Saveetha Dental College and Hospitals, Saveetha Institute of Medical and Technical Sciences, Saveetha University, Chennai - 600 077, India; 2 Department of Biotechnology, Government Science College, Chitradurga-577501, Karnataka, India; 3 Department of Biochemistry, Saveetha Dental College and Hospitals, Saveetha Institute of Medical and Technical Sciences, Saveetha University, Chennai - 600 077, India; 4 Department of Stem Cell Biology, Stellixir Biotech Pvt Ltd, No.V-31, 2nd floor, 10th Main Road, Peenya 2nd Stage Industrial Area, Bangalore - 560058, Karnataka, India; 5 Department of Biochemistry and Department of Clinical Skills and Simulation, Panimalar Medical College Hospital and Research Institute, Varadharajapuram, Poonamallee, Chennai - 600 123, India; 6 Exonn Biosciences, Ticel Bio Park, Chennai-600 0113, India;; 7 PG and Research Department of Biotechnology and Bioinformatics, Holy Cross College (Autonomous), Trichy- 620002, Tamil Nadu, India; 8 Central Research Laboratory, Meenakshi Academy of Higher Education and Research (Deemed to be University), Chennai-600 078, India

**Keywords:** Alzheimer's disease, Protein Network analysis, Gymnema sylvestre, Molecular docking

## Abstract

Amyloid precursor protein is linked with Alzheimer's disease (AD). The Australian cowplant Gymnema sylvestre is known in Indian and Chinese medicine. Therefore, it is of interest to screen the Amyloid precursor protein with compounds from the Australian cowplant. We report five compounds (Gymnemasaponin 5, Gymnemasin D, Gymnemoside A, Gymnemoside E, Gymnemoside F) derived from the Australian cowplant as the poteinal inhibitors of Amyloid precursor protein with optimal binding features for further consideration.

## Background

Alzheimer's disease (AD) is the most frequent form of neurodegenerative disorder and the most prevalent cause of dementia commonly affecting the elderly people. It is well known that AD has complex various pathogenic factors, for example genetic factor, environmental factor, immunological factor, head injuries, depression, or hypertension [1- 5]. Among these factors, genetic factors are probable to attribute about 70% to the danger for AD [6]. As well, genetic analyses have confirmed that, personality differences of AD could be resulted from many genes and their variants, which apply a variety of biological functions in coordination to improve the risk of the disease [7,8,9]. Except for spot out the mechanisms drawn in in the AD pathogenesis, wide-ranging analyses of possible candidate genes could propose novel possible strategies to predictive or diagnostic test for AD. Hence in the present study we aimed to construct the current experimentally supported network of direct human protein interactions, explore it for potential target proteins for AD. Gymnema sylvestre is (commonly named as Gurmar, family: Asclepiadaceae) is one of the well known plant used in diabetic conditions and is officially mentioned in Indian Pharmacopoeia [10]. This plant has been proved for its various medicinal properties like anticancer, antimicrobial, antiarthritic [11]. Therefore, it is of interest to screen the Amyloid precursor protein with compounds from the Australian cow plant.

## Materials and Methods:

### Protein network constructions and hub identification:

Alzheimer's disease related protein network was constructed in cytoscape software [12]. Hub proteins were selected using the cytohubba plugin software [13].

### Ligand Preparation:

Known compounds from Gymnema sylvestre was collected from literature. The corresponding data was downloaded from pubchem database in SDF and converted to the PDB format using Online Smiles Translator. Energy minimizations of ligands were done using ChemBio 3D Ultra 12.0.

### Target protein:

High Resolution crystallographic structure of Amyloid beta protein (PDB ID: 1AAP) was downloaded from the RCSB PDB database [14].

### Molecular Docking analysis:

The PatchDock server [15,16] was used for the docking of Known compounds from Gymnema sylvestre was collected from literature with the Amyloid beta protein (PDB ID: 1AAP).

## Results and discussion:

**Figure 1:**
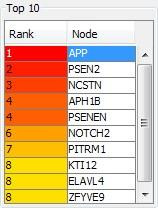
Hub proteins Identified from Cytohubba.

**Figure 2:**
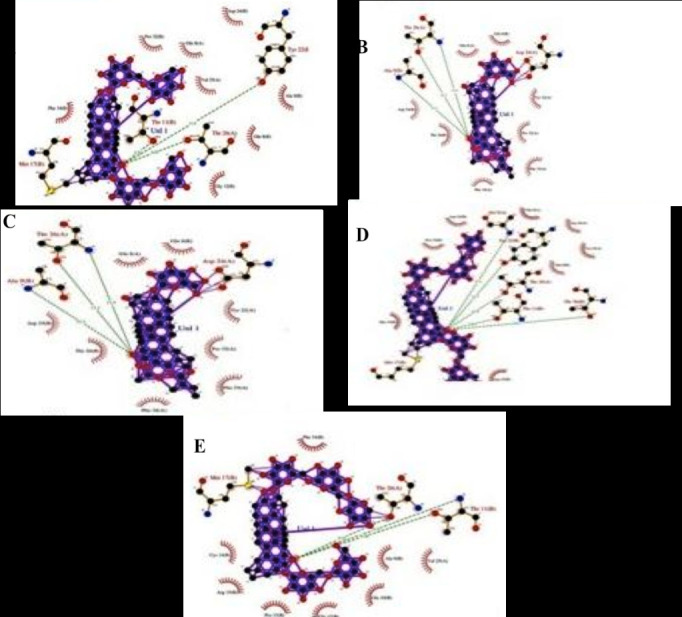
Ligplot analysis of docked amyloid precursor protein in complex with (A) Gymnemasaponin 5; (B) Gymnemasin D; (C) Gymnemoside A; (D) Gymnemoside E; (E) Gymnemoside F.

In the present study Hub protein identification was performed using cytohubba. Cytohubba results were calculated based on Maximal Clique Centrality algorithm. Ten proteins were identified as most interactive proteins in the network. Among ten, the top most protein with Rank 1 was Amyloid precursor protein (Figure 1). It is an integral membrane protein expressed in many tissues and concentrated in the synapses of neurons. This Amyloid precursor protein is considered as a potential target for Alzheimer's disease. Hence in the present study it was taken as target protein for molecular docking studies. Compounds from Gymnema sylvestre was used for docking studies with Amyloid precursor protein and the interaction were analyzed. In the molecular docking paradigm, the ligand with more negative binding energy is considered more successful and more potent than to the one with less or positive binding energy. In the present study, results from PatchDock analysis revealed that the binding affinity of selected natural compounds gives significant result. For this study we took 26 phytocompounds from Gymnema sylvestre. Based on the results of docking studies best five were shown in Table 1. The atomic contact energy (ACE) value of selected 5 compounds range from -638.16 to -236.92 Kcal/mol. Thus from the calculated ACE values it can be inferred that these compounds showed the favorable binding energy with Amyloid precursor protein. This ligplot analysis shows the hydrogen bonding and the length of their interaction with the key residues of the Amyloid precursor protein in the active site pocket. Among the residues which play a vital role in the mechanisms of action we found that the ALA 9, THR11, TYR22, THR26 are the main interacting amino acids residues (Figure 2).

## Conclusions:

We report five compounds (Gymnemasaponin 5, Gymnemasin D, Gymnemoside A, Gymnemoside E, Gymnemoside F) derived from the Australian cowplant as poteinal inhibitors of the Amyloid precursor protein with optimal binding features for further consideration.

## Figures and Tables

**Table 1:** Molecular docking results obtained through Patch dock server

S. No	Compound name	Score(kca l/mol)	ACE	Hond interaction	H bond length	No of non-bonded interaction
1	Gymnemasaponin 5	5398	-408.46	THR 26 OG1-O	2.51	144
				TYR 22 OH-O	2.29	
2	Gymnemasin D	3790	-236.92	ALA 9 N-O	3.04	156
				THR 26 N-O	2.83	
				THR 26 OG1-O	2.69	
3	Gymnemoside A	4668	-311.8	ALA 9 N-O	2.69	142
				THR 26 OG1-O	1.92	
				THR 26 N-O	2.83	
4	Gymnemoside E	5850	-358.55	ALA 9 N-O	2.74	147
				THR 26 OG1-O	3.01	
				THR 11 OG1-O	2.72	
				TYR 22 OH-O	2.65	
				THR 26 OG1-O	3.18	
5	Gymnemoside F	5384	-638.16	THR 26 OG1-O	2.09	263
				THR 11 N-O	3.13	
				THR 11 OG1-O	2.19
